# Laparoscopic repair for neonatal spontaneous gastric perforation: a case report

**DOI:** 10.3389/fsurg.2025.1735389

**Published:** 2025-12-11

**Authors:** Jianqing Wu, Lin Ye, Xiqin Wang, Heng Zhang

**Affiliations:** 1 Department of Pediatric Surgery, Lishui Central Hospital, The Fifth Affiliated Hospital of Wenzhou Medical University, Lishui, China; 2 Department of Central Laboratory, Lishui Central Hospital, The Fifth Affiliated Hospital of Wenzhou Medical University, Lishui, China

**Keywords:** neonate, gastric perforation, congenital gastric wall defect, laparoscopy, minimally invasive surgical procedures

## Abstract

**Background:**

Spontaneous neonatal gastric perforation (SNGP) is an extremely rare but life-threatening surgical emergency. Open surgery has been the traditional mainstay of treatment, whereas reports on laparoscopic repair in neonates remain scarce. This report delineates a successful case of laparoscopic repair for SNGP in a newborn. We herein detail the surgical technique and elucidate the advantages of this minimally invasive approach over conventional open surgery.

**Case presentation:**

A 34-week premature male neonate, with a birth weight of 2550 grams and born to a G6P4 mother, was admitted presenting with a one-day history of vomiting and abdominal distension. An abdominal x-ray obtained at a referring hospital demonstrated pneumoperitoneum, suggestive of neonatal gastrointestinal perforation. Upon transfer to our institution, a repeat abdominal x-ray revealed massive subdiaphragmatic free air, elevated diaphragmatic arches, and central clustering of bowel loops, constituting the classic “football sign.” A preliminary diagnosis of spontaneous neonatal gastric perforation was established. The patient subsequently underwent emergent laparoscopic repair of the gastric perforation. Postoperatively, he was transferred to the neonatal intensive care unit (NICU) for close monitoring. Full enteral feeding was gradually reestablished, commencing on the seventh postoperative day.

**Conclusion:**

In this case, a newborn with SNGP was successfully managed via laparoscopic repair. This case underscores the potential of minimally invasive techniques for managing this condition. The laparoscopic approach provided superior visualization of the gastric fundus and facilitated enhanced vessel mobilization compared to open surgery. The patient's uneventful postoperative recovery exemplifies the documented advantages of laparoscopy, including reduced tissue trauma, diminished postoperative pain, and a more rapid recovery. We therefore conclude that, for hemodynamically stable neonates in centers with appropriate surgical expertise, laparoscopic repair represents a viable and advantageous alternative to open surgery.

## Introduction

Spontaneous neonatal gastric perforation (SNGP) is a rare but life-threatening abdominal emergency, accounting for approximately 7% of all neonatal gastrointestinal perforations, with a higher incidence in preterm infants. Most cases manifest between the second and seventh day of life. Although the initial presentation can be subtle, the disease progression is often rapid and associated with high mortality (1, 2). The condition was first described by Siebold in 1826 (3). The etiology of neonatal gastric perforation is broadly categorized into traumatic, ischemic, and spontaneous types (4). Although the precise pathogenesis remains incompletely elucidated, several pathogenic mechanisms have been proposed. Postulated mechanisms include congenital muscular defects, ischemic necrosis due to impaired perfusion, stress- or infection-related mucosal erosion, and elevated intragastric pressure secondary to non-invasive ventilation or excessive air swallowing (5). Typical clinical manifestations of SNGP encompass sudden abdominal distension, respiratory distress, and circulatory failure. An abdominal plain film often reveals pneumoperitoneum, presenting the classic “football sign” (6). Emergency exploratory laparotomy has been the conventional surgical intervention. However, this procedure is highly invasive and poses a significant physiological burden on critically ill neonates. This article presents a case of SNGP that was successfully managed laparoscopically, and discusses the feasibility, key technical considerations, and potential benefits of this minimally invasive approach in high-risk neonates, aiming to provide insights for clinical management.

## Case presentation

**Figure 1:**
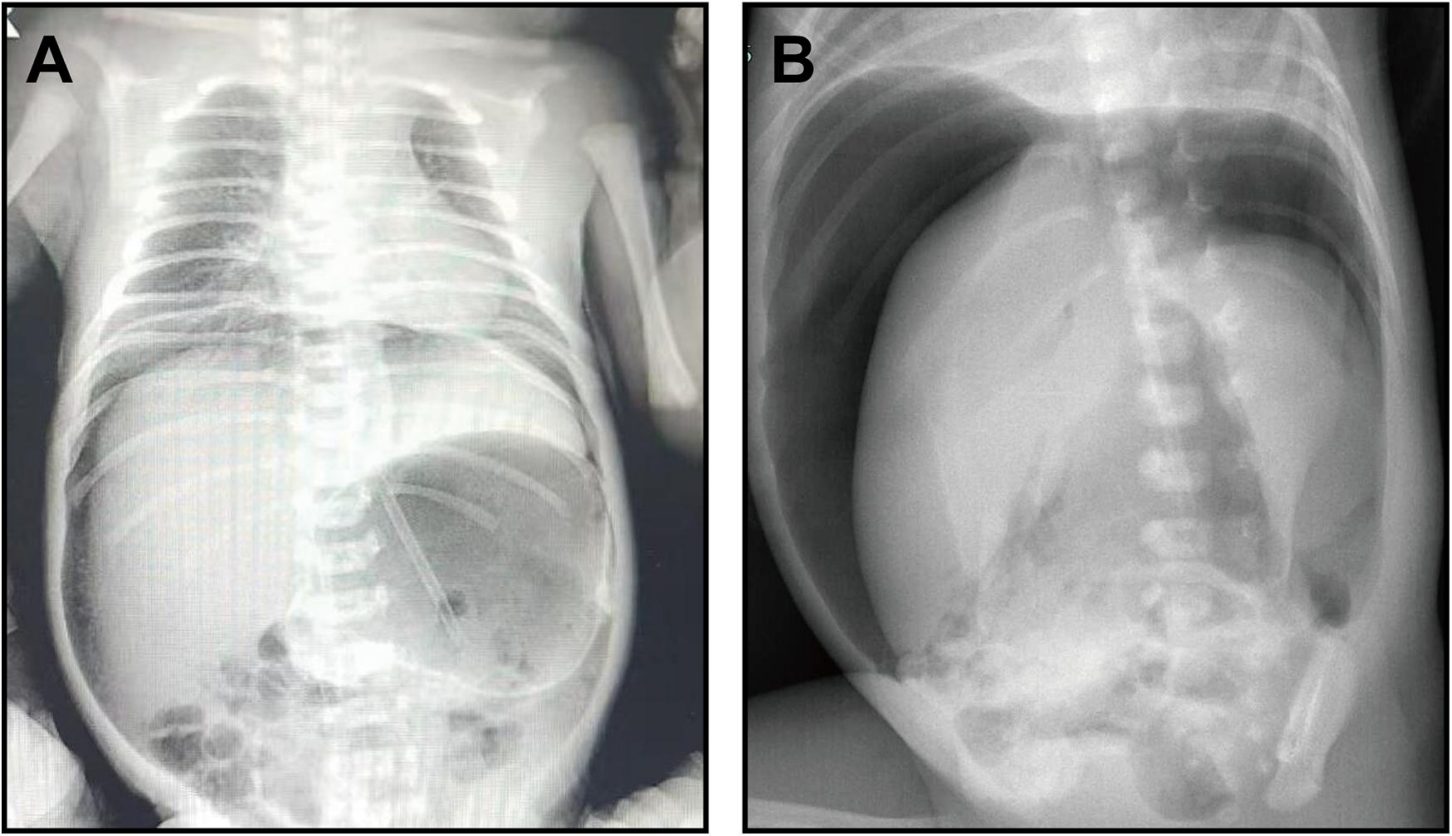
Preoperative abdominal radiographs demonstrating pneumoperitoneum. **(A)** An initial abdominal radiograph obtained at a referring hospital, revealing pneumoperitoneum. **(B)** A follow-up radiograph upon admission to our center, showing massive subdiaphragmatic free air and the classic “football sign”, indicative of spontaneous neonatal gastric perforation (SNGP).

A 34-week preterm female infant, the smaller of twins with a birth weight of 2,550 grams, was delivered via cesarean section. The mother (G6P4) had an uncomplicated pregnancy with normal prenatal screenings. The infant had Apgar scores of 10 at 1 and 5 minutes. Shortly after birth, she developed moaning and tachypnea, necessitating admission to the neonatal unit for respiratory support and preterm care. Following feeding initiation, she exhibited recurrent non-bilious vomiting and gastric retention. Despite fasting and gastric decompression, she developed progressive abdominal distension. An abdominal x-ray revealed pneumoperitoneum (Figure 1A), leading to a diagnosis of neonatal gastrointestinal perforation and prompt transfer to our neonatal center for specialized management.

Upon admission, the infant was stable with a strong cry and normal responsiveness. Physical examination revealed a flat anterior fontanelle, an *in situ* gastric tube, and a distended abdomen with visible venous patterning, muscular rigidity, and absent bowel sounds. Extremities were warm with normal tone. Emergency laboratory investigations, including complete blood count, CRP, biochemistry, electrolytes, coagulation profile, and procalcitonin, were within normal limits. An emergency abdominal x-ray demonstrated crescent-shaped subdiaphragmatic free air and the classic “football sign” of centrally clustered bowel loops (Figure 1B), confirming the preliminary diagnosis of spontaneous neonatal gastric perforation.

**Figure 2:**
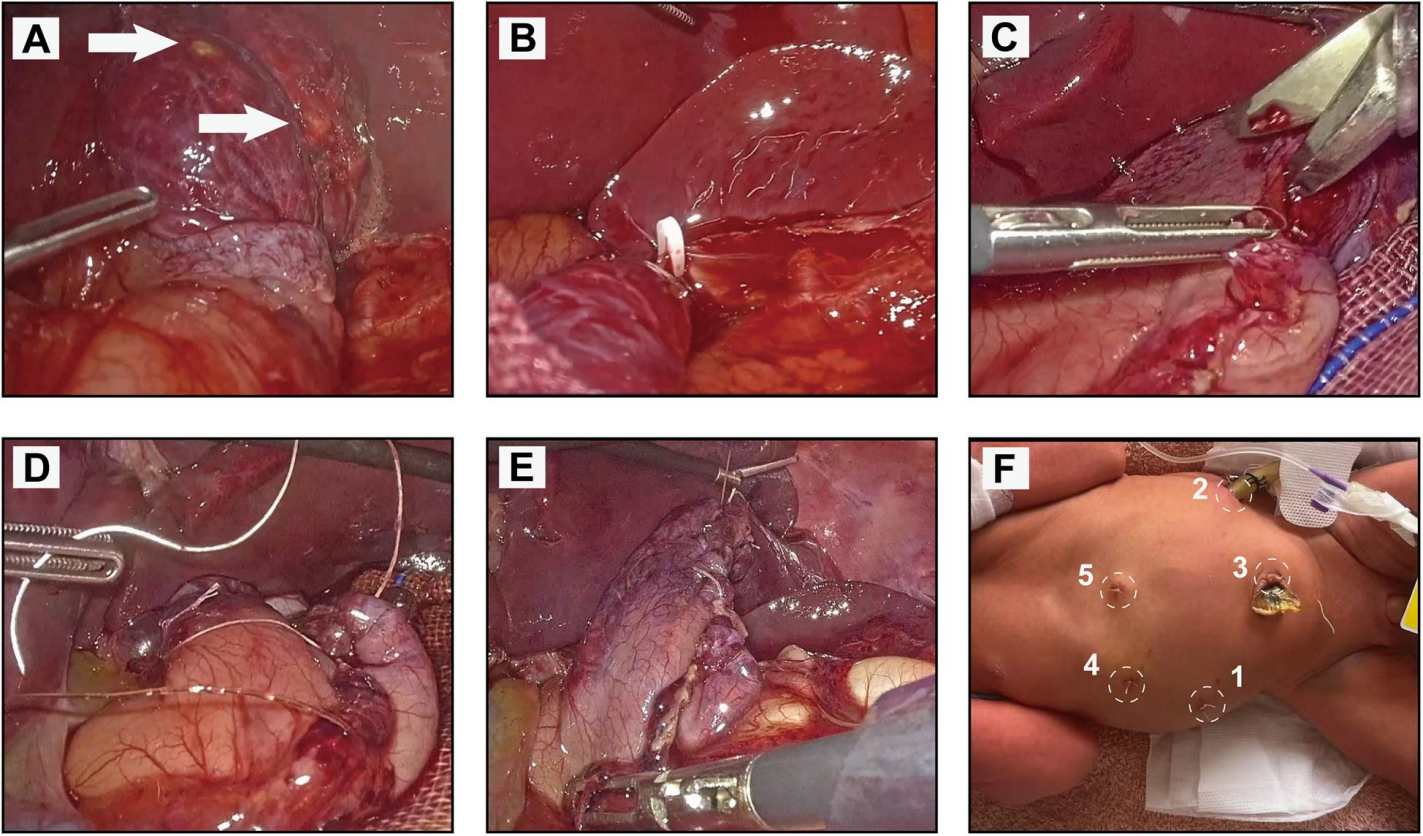
Sequential intraoperative views of the laparoscopic repair for spontaneous neonatal gastric perforation. **(A)** Intraoperative inspection identified extensive serosal tearing and muscularis defects, featuring two perforations (approximately 2 mm, arrows) surrounded by purulent exudate. **(B)** Isolation and clamping of the short gastric vessels using Hemlock forceps, with careful preservation of the posterior gastric vessels. **(C)** Resection of the non-viable gastric tissue and the muscularis defect using laparoscopic scissors. **(D)** Continuous suture closure of the gastric defect along the greater curvature with 4-0 barbed suture. Reinforcement of the suture line with interrupted 5-0 VICRYL sutures. **(E)** Final appearance of the repaired stomach following suture reinforcement. **(F)** Postoperative schematic of trocar placements: 1, secondary working port; 2, primary working port; 3, camera port; 4, assistant port; 5, liver retractor port.

Given the patient's stable vital signs, hemodynamic stability, and tolerance for pneumoperitoneum, we decided to proceed with emergency laparoscopic surgery. Preoperative preparation included nasogastric and urinary catheterization, availability of packed red blood cells and plasma, and establishment of central and peripheral venous access. Preoperative vital signs were stable: temperature, 36.5 °C; respiratory rate, 48 breaths/min; heart rate, 168 bpm; blood pressure, 77/51 mmHg; and oxygen saturation, 99%. Under combined intravenous-inhalational anesthesia in the supine position, pneumoperitoneum was established via a 10 mm umbilical trocar with CO_2_ insufflation at 6 mmHg pressure and 20 L/min flow. A 3D STROZ laparoscope revealed a large muscular layer defect along the greater curvature with extensive serosal tearing, featuring two approximately 2 mm perforations surrounded by purulent exudate (Figure 2A).

Laparoscopic repair was initiated with additional ports: a 5 mm port in the left lower mid-abdomen (primary working port), 3 mm ports below the right costal margin, in the right lower mid-abdomen, and a subxiphoid port for liver retraction (Figure 2F). The gastroepiploic pouch was opened to expose the greater curvature. Short gastric vessels were divided with preservation of posterior gastric vessels (Figure 2B). After demarcating lesion boundaries with methylene blue, devitalized tissue was resected using laparoscopic scissors (Figure 2C). The gastric defect was closed with continuous 4-0 barbed suture, reinforced with interrupted 5-0 VICRYL sutures (Figure 2D). Hemostasis was confirmed, and an air leak test via nasogastric tube showed no leakage (Figure 2E). Resected tissue was retrieved through the 5 mm port for pathological examination. After saline irrigation until clear, hemostatic material was applied, and a drainage tube was placed via the left lower port. Skin incisions were closed with estimated blood loss of 20 mL, replaced with packed red blood cells. The laparoscopic procedure was completed uneventfully in 47 min from incision to closure.

Postoperative NICU management included broad-spectrum antibiotics, gastrointestinal decompression, and total parenteral nutrition. Drain outputs progressively decreased. A gastrointestinal contrast study on postoperative day 7 confirmed complete healing without leakage, allowing removal of gastric and drainage tubes. Initial feeding attempts with standard formula resulted in intolerance (rash and reflux), which resolved after switching to amino acid-based formula. Full enteral feeding was achieved by day 17, followed by transfer to rooming-in and discharge on day 20. At discharge, the infant demonstrated good weight gain, healed incisions, and excellent formula tolerance. Pathological examination confirmed congenital gastric musculature defect with inflammatory changes at the perforation margins. One-month follow-up showed satisfactory weight gain without gastrointestinal symptoms.

## Discussion

This report demonstrates the successful laparoscopic management of spontaneous gastric perforation in a newborn. Timely intervention is paramount in such cases, as prompt recognition and surgical treatment represent the primary determinants of survival in neonatal abdominal emergencies (7). Diagnostic or therapeutic delays substantially increase the risks of shock, sepsis, and multi-organ failure, with corresponding increases in mortality. Thus, early diagnosis, decisive clinical decision-making, and expedited surgery are crucial for improving prognosis (8).

While conventional open laparotomy remains the traditional approach for neonatal gastric perforation, it is associated with significant tissue trauma, pronounced postoperative pain, prolonged recovery, extended hospitalization, and elevated risk of surgical site infection. This case illustrates that laparoscopic repair is a feasible, safe, and effective alternative in regional neonatal critical care centers. The laparoscopic approach offers several distinct advantages: (1) Enhanced visualization and precision—laparoscopic magnification provides superior assessment of the perforation site and extent, particularly improving exposure of the gastric fundus and visualization of posterior and short gastric vessels compared to open surgery; (2) Minimally invasive benefits—including smaller incisions, reduced postoperative pain, accelerated recovery, superior cosmesis, minimized visceral manipulation, and decreased adhesion formation (9, 10); (3) Diagnostic capability—when clinical suspicion of perforation exists without clear localization, comprehensive laparoscopic exploration can be performed (11); (4) Educational value—the procedure can be recorded for academic exchange, training, and research purposes.

General anesthesia is the standard of care for neonatal surgery. A successful anesthetic outcome depends on a multifaceted strategy encompassing comprehensive preoperative evaluation, anticipatory preparation, precise intraoperative monitoring, lung-protective ventilation, goal-directed hemodynamic support, and meticulous temperature management (12). The principal anesthetic challenge involved maintaining adequate oxygenation, ventilation, and circulatory stability amidst the inherent pathophysiological disturbances of the condition and the additional risks posed by CO_2_ pneumoperitoneum, such as hypercapnia, gas embolism, pneumothorax, and pneumomediastinum. A thorough preoperative assessment of the infant's systemic and respiratory status was conducted. Anesthesia was maintained with a balanced intravenous-inhalational technique via endotracheal intubation and controlled ventilation. Pneumoperitoneum was established and maintained at a pressure of 6 mmHg and a flow rate of 20 L/min. Intraoperative anesthesia management was uneventful, and the patient was successfully extubated at the conclusion of the procedure. Given the limited abdominal domain in neonates, we utilized 3 mm Trocars and exercised extreme precision in instrument manipulation to avoid iatrogenic injury. Systematic exploration of the entire gastrointestinal tract is essential to identify potential concomitant perforations or anomalies (13). Postoperative drainage placement adjacent to the repair site provides critical monitoring for anastomotic integrity and ensures adequate fluid evacuation.

Neonates with gastrointestinal perforation often experience rapid clinical deterioration, with high susceptibility to circulatory failure, acidosis, and respiratory compromise. Early diagnosis and comprehensive supportive care are therefore essential for optimal outcomes (7). Thorough preoperative preparation is particularly important given the reduced physiological reserve in this population. Laparoscopic repair of gastric perforation presents unique technical challenges, as the pathological process frequently involves extensive gastric segments, potentially necessitating total, subtotal, or partial gastrectomy (14). This procedure requires advanced laparoscopic skills and specialized instrumentation, and should only be undertaken in appropriately equipped centers with surgical expertise (15, 16). Laparoscopic repair is a viable and advantageous option for hemodynamically stable neonates with gastric perforation. A fundamental prerequisite for this procedure is the patient's capacity to tolerate the cardiorespiratory effects induced by CO_2_ pneumoperitoneum. When neonates present with hemodynamic instability or shock that fails to respond to initial resuscitation with anti-infectives and decompression, exploratory laparotomy becomes necessary. In such cases, it remains the optimal and most expeditious surgical choice. Furthermore, laparoscopy is contraindicated in cases of severe cardiac or pulmonary compromise, coagulopathy, or pre-existing hemodynamic instability (12).

## Conclusions

Spontaneous neonatal gastric perforation (SNGP) represents a rare yet rapidly progressive surgical emergency. Timely surgical intervention is crucial for effective management. In hemodynamically stable neonates with gastric perforation, laparoscopic repair emerges as a valuable minimally invasive alternative in centers possessing the requisite surgical expertise and specialized equipment. The favorable outcome in this case substantiates the feasibility of this technique and serves as a reference for its clinical application. Nonetheless, broader implementation warrants further validation through larger case series and long-term follow-up studies.

## Data Availability

The original contributions presented in the study are included in the article/Supplementary Material, further inquiries can be directed to the corresponding author/s.
